# THE RELATIONSHIP BETWEEN SOCIAL NETWORK AND PURPOSE IN LIFE: APPLICATION OF A RANDOM INTERCEPT CROSS-LAGGED MODEL

**DOI:** 10.1093/geroni/igac059.2640

**Published:** 2022-12-20

**Authors:** Gina Lee

**Affiliations:** Iowa State University, Ames, Iowa, United States

## Abstract

The purpose of this study is to examine if there are reciprocal effects between social network size and purpose in life among older adults using data from the National Health and Aging Trends Study. A second aim is to assess whether there are moderated effect of gender on this relationship. The sample included 1,485 male and 2,058 female adults 65 years and older. In order to examine the reciprocal effects between social network size and purpose in life over four time points (2017, 2018, 2019, and 2020), a random intercept cross-lagged model (Model 1) was computed. Then, two multiple group RI-CLPM analyses (Model 2 and 3) were computed in order to test moderation of gender on the relationship. Model 2 estimates the cross-lagged parameters freed and model 3 estimates the cross-lagged parameters constrained. The results indicated that Model 1 fit the data well, χ2(9) = 26.06, p = .002. The carry-over effects of social network and purpose in life were significant. The spill-over effect from wave 3 purpose in life on wave 4 social network was significant. The variance of the random intercepts were significant, indicating that there are stable, trait-like differences between social network and purpose in life. The results of the multiple group model revealed that there were no significant differences between the freed and constrained models, indicating that lagged effect for male and female appear to be same. Future research should explore if other factors like race or education show moderated effects.

